# Effect of gingival biotype on orthodontic treatment-inducedperiodontal complications: A systematic review

**DOI:** 10.34172/japid.2020.003

**Published:** 2020-04-14

**Authors:** Reza Amid, Mahdi Kadkhodazadeh, Anahita Moscowchi, Shiva Tavakol Davani, Milad Soleimani, Anahita Dehghani Soltani, Muna Al-Shuhayeb

**Affiliations:** ^1^Dental Research Center, Research Institute of Dental Sciences, School of Dentistry, Shahid Beheshti University of Medical Sciences, Tehran, Iran; ^2^Department of Periodontics, School of Dentistry, Shahid Beheshti University of Medical Sciences, Tehran, Iran; ^3^Department of Orthodontics, School of Dentistry, Shahid Beheshti University of Medical Sciences, Tehran, Iran

**Keywords:** Gingival biotype, gingival recession, gingival thickness, orthodontics

## Abstract

**Background:**

It is crucial to maintain periodontal health in patients undergoing orthodontic treatment. Biotype is a critical factor to be considered in this regard. This systematic review investigated the scientific evidence on the relationship between gingival biotype and marginal periodontal alterations induced by orthodontic interventions.

**Methods:**

An electronic search was conducted for pertinent studies in three databases: PubMed, Scopus, and Cochrane up to August 1, 2019 based on a detailed protocol according to the PRISMA statement. The authors also completed a hand search in six dental journals and the bibliographic lists of the relevant studies.

**Results:**

Of 1512 citations retrieved through the electronic search, 602 were duplicate entries. By evaluating titles, abstracts, and full texts, eight articles conformed to the inclusion criteria; however, no relevant studies were found through hand searching. The evidence suggested that recession was inversely related with the thickness of the facial margin. These findings were more evident in proclined teeth and patients using fixed appliances.

**Conclusion:**

The existing evidence suggests that orthodontic therapy might result in mild detrimental effects on the periodontium, especially in patients with thin biotype. However, due to the limited investigations and their inconsistent methodology, further well-designed prospective studies are necessary.

## Introduction

Malocclusion is the third most prevalent oral health problem worldwide.^1,2^ Orthodontic treatments facilitate oral hygiene measures and establish occlusal stability and lip competency by eliminating traumatic occlusion and crowding; thus, some investigators have considered these interventions as potential means to improve periodontal health.^3,4^ However, orthodontic appliances might increase plaque accumulation and impede proper oral hygiene, which raise the possibility of making these treatments detrimental to periodontal tissues.^5-9^

Lindhe and Seibert^10^ used the term “periodontal biotype” to describe morphologic characteristics of the periodontium. In general, there are two types of gingival biotype: “thin scalloped” and “thick flat.”^11,12^ The biotype depends on many factors, including age, sex, genetic factors, as well as the shape, position, and size of the teeth.^13^ In addition, the width and thickness of the facial gingiva vary from one individual to another, and even in different regions of a mouth. Therefore, there are diverse “gingival phenotypes,” a term used by Muller and Eger^14^ for the first time. Studies have shown that gingival thickness plays a fundamental role in mucogingival problems. As the attachment level is minimal in thin biotype, it is more prone to trauma and inflammation.^15-17^ Consequently, accurate pre-orthodontic evaluation of the biotype has been recommended in order to preclude potential complications.^17-19^

Given the increasing demand for orthodontic treatments and the importance of maintaining periodontal health, the nature and extent of the complications related to these interventions in patients with different biotypes should be taken into account. Despite the conflicting opinions about the relationship between orthodontic treatments and periodontal health, few studies have addressed the orthodontic-related changes affecting marginal periodontal tissues. This study investigated the scientific evidence on the relationship between gingival biotype and periodontal changes caused by orthodontic movements.

## Methods

### Protocol

A detailed protocol was developed and followed, according to the PRISMA statement.^20,21^

### Search strategy

The eligibility criteria were as follows:

-Study design: We evaluated randomized controlled trials (RCTs) and prospective, retrospective, and cross-sectional studies.

-Population: We included only studies on humans, with no restrictions in terms of patient’s age or occlusion characteristics, although we did exclude studies that included patients with severe periodontal diseases or craniofacial anomalies.

-Intervention: We focused on studies assessing fixed or removable orthodontic appliances, or both. Since orthognathic surgery and distraction osteogenesis might have different consequences compared to nonsurgical orthodontic therapy, we agreed to exclude studies comprising these procedures.

-Comparison: We assessed periodontal outcomes in patients with different types of gingival biotype, who underwent orthodontic treatment.

-Types of outcome measures: Owing to the heterogeneity of endpoints in periodontal studies,^22^ we could formulate no single periodontal outcome measure. Instead, we included all studies with at least one type of periodontal parameter.

### Search methods

Two authors (RA and AM) extracted articles through an electronic search and a hand search of the specific journals. The reference lists of the selected full-text articles were also screened for the unidentified or unpublished relevant studies.

-Electronic search: PubMed, Scopus, and the Cochrane Library (including the Cochrane Central Register of Controlled Trials [CENTRAL] and the Cochrane Database of Systematic Reviews [CDSR]) were searched up to August 1, 2019. We did not limit our search strategy regarding the study design, as doing so could have excluded some pertinent publications.^23^ No publication status and language or time restrictions were applied.

Search terms included the following keywords and were modified appropriately for each database.

Search #1: Orthodontic* AND (“gingival biotype” OR “periodontal biotype” OR “gingival thickness”)

Search #2: Orthodontic* AND (“gingival recession” OR “gingival side effect” OR “periodontal side effect” OR mucogingival)

Hand search: Five journals, American Journal of Orthodontics and Dentofacial Orthopedics, Angle Orthodontist, Journal of Periodontology, Journal of Dental Research and Journal of Clinical Periodontology, were hand-searched for studies reporting on the periodontal effects of orthodontic treatment in patients with different types of gingival biotype.

The titles and abstracts of the retrieved articles were screened independently by two authors (RA and AM) to assess the fulfillment of the inclusion criteria. Full-text articles were obtained in case the supplementary data were needed. Any disagreements during the process were resolved by discussion.

### Quality assessment

Two of the reviewers (RA and MK) independently assessed the quality of the identified studies and resolved any disagreements through discussion.

For cohort, case–control and cross-sectional studies, we used the Newcastle-Ottawa Quality Assessment Scale, which consists of eight items: four items regarding selection, one item regarding the comparability of the groups, and three items regarding the outcome assessment.^24^ Then, we classified the bias status as low (all quality items met), moderate (one or two quality items not met), or high (three or more criteria not met).

Data Extraction and Synthesis

Four reviewers (STD, MS, ADS, and MAS) independently extracted the relevant data by using a predefined data extraction table to report on the study design, participants, orthodontic intervention, periodontal outcomes, and the gingival biotype.

## Results

### Search results

**Figure 1:**
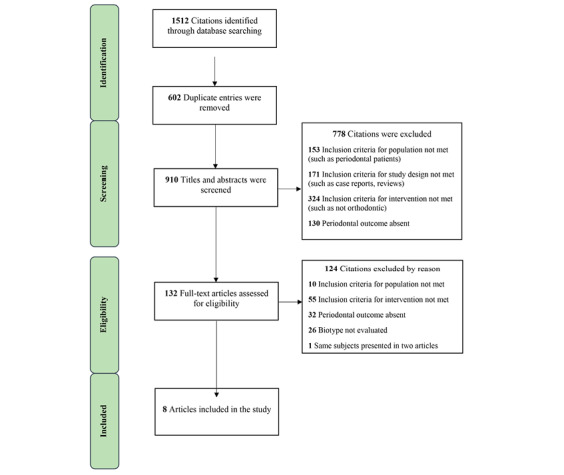
The flowchart of the study selection process.

Of the 1512 citations retrieved through the electronic search, 602 were duplicate entries. Eight articles were eligible to be included in the study through evaluation of the titles, abstracts, and full texts (Figure 1). We found no studies that met the inclusion criteria through the hand searching of the literature. The characteristics of the included studies are presented in Table 1.

**Table 1:** The characteristics of the included studies

**Characteristic**	**Study**							
	**Ji et al (28)**	**Stappert et al (27)**	**Rasperini et al (16)**	**Boke et al (8)**	**Yared et al (25)**	**Szarmach et al (30)**	**Melsen, Allias (29)**	**Ngan et al (26)**
**Study Design**	**Retrospective**	**Prospective**	**Prospective**	**Retrospective**	**Retrospective**	**Cross-sectional**	**Retrospective**	**Retrospective**
**Participants**	403 (285 F, 118 M)	29	16 (6 F, 10 M)	251 (177 F, 74 M)	34	18 (11 F, 7 M)	150 (114 F, 36 M)	20 (12 F, 8 M)
**Age**	11–43	≤13 14–18 ≥19	Mean age: 21±8.20	8-17.8 (mean 13.37 ± 2.06)	18-33	12-39	F: 22-65 M: 23-50	11-16
**Biotype determination**	Probing transparency	Transgingival probing	Biotype probe (Hu-Friedy)	Intra-oral photographs, Visual inspection of the gingival texture and capillary transparency	Scaled digital caliper,	Periodontal probe	Intra-oral photographs, Visual inspection of the gingival texture and capillary transparency	Visual inspection
**Biotype classification**	Thin Thick	Thin (≤2.5mm) Thick (>2.5mm)	Thin Medium Thick Very thick	Thin Thick	<0.5 mm ≥0.5 mm	Thin gingiva and the root bone layer	Thin Thick	Thin Moderate Thick
**Intervention**	NM	Fixed appliance with premolar extraction	Fixed appliance	58 Fixed appliance with extraction 173 Fixed appliance without extraction 20 Functional appliance	Fixed appliance	Fixed appliance	Fixed appliance without extraction	NM
**Periodontal Outcome**	GI, PI, GR, and plaque index	Gingival cleft	PPD, GR, CAL, KTW, FMBS	GR, Plaque, Inflammation	Plaque index, GI, PPD, CAL, GR	GR	GR, Plaque, Inflammation	GR, Inflammation
Quality assessment*	***	****	******	***	***	***	***	****

F = Female, M = Male, GI = Gingival Index, PI = Periodontal Index, GR = Gingival Recession, PPD = Probing Pocket Depth, CAL = Clinical Attachment Level, KTW = Keratinized Tissue Width, FMBS = Full-mouth Bleeding Score NM = not mentioned *Number of the stars represents quality of the study based on the Newcastle-Ottawa Quality Assessment Scale

The participants were 8–65 years of age, with the majority undergoing orthodontic treatment as adolescents or young adults. They had various forms of malocclusion. Three studies^25-27^ did not report the malocclusion status. One study restricted the inclusion criteria to those who had infraversion or open bite,^28^ and one study included only subjects with Class I and Class II malocclusion.^29^ The types of orthodontic treatments were fixed appliances,^8,16,25,29,30^ fixed appliances with premolar extraction,^8,27^ functional appliances,^8^ or not reported.^26,28^

The selected articles reported various markers of periodontal status as follows: gingival recession,^8,16,25,26,28-30^ probing pocket depth,^16,25^ clinical attachment level,^16,25^ inflammation,^8,16,25,26,28,29^ periodontal index,^28^ plaque index,^8,25,28,29^ and keratinized tissue width.^16,25^ One study reported gingival clefts,^27^ but none of them reported on tooth loss, tooth mobility, or other adverse effects. In addition, Szarmach et al^30^ evaluated crowding, protrusion, improper frenal attachment, and the depth of the oral vestibule.

Regarding gingival biotype determination, Rasperini et al,^16^ Melsen and Allais,^29^ and Ngan et al^26^ used the four mandibular incisors, while Yared et al^25^ evaluated the mandibular central incisors.

The results of these investigations indicated that some periodontal complications, such as increased probing depth, attachment loss, and gingival recession might be more prevalent in orthodontic patients; five studies^16,25,28-30^ reported that recession was inversely related with the width of keratinized gingiva and gingival thickness. Rasperini et al16 demonstrated that the thin biotype and incisor proclination could induce gingival recession (0.17 mm) and reduce the width of keratinized gingiva (-0.67 mm), which was not evident in the alignment and retroclination movements. However, only one of their patients showed a gingival recession of 1.5 mm on a mandibular left central incisor. Their study also revealed no significant relationship between the biotype and changes in probing depth and attachment loss.

Boke et al^8^ reported that in patients treated with fixed appliances, the mean values of visible plaque, visible inflammation, and gingival recession were 2.93±6.78, 2.76±6.20, and 0.11±0.40 before treatment, respectively. These parameters increased significantly after orthodontic treatment and reached 5.92±9.08, 17.75±18.74, and 0.48±1.13, respectively; however, such a relationship was not evident for functional appliances. The canines were the most affected teeth by gingival recession (9.48% for maxillary and 7.76% for mandibular canines in the extraction group; 4.04% for maxillary and 3.76% for mandibular cuspids in the non-extraction group).

Szarmach et al^30^ reported that cases with thin gingival margin and thin buccal bone had more recession, which was evident more frequently in skeletal class III patients. Melsen and Allais^29^ demonstrated that only 2.8% of the subjects developed recession >2 mm, and 5% of the pre-existing gingival recessions improved. They concluded the baseline recession, width of keratinized gingiva, gingival biotype, and gingival inflammation were correlated with gingival recession.

## Discussion

Orthodontic appliances might damage periodontal tissues by creating retentive areas for dental plaque; even with excellent oral hygiene, the appliances cause a change in the intraoral microflora, leading to a bacterial array similar to that present in sites affected by periodontal disease.^31^

In contrast to patients with thick gingiva, those with a thin-scalloped biotype are considered at risk; therefore, identification of these high-risk subjects is warranted. The evidence identified by this systematic review suggested that there might be an association between the gingival biotype and orthodontic treatment-induced periodontal complications.^16,25,27-30^ However, there are some limitations, including a limited number of studies, potential of bias, inconsistent methods for biotype determination, and various orthodontic interventions, making the comparison of the results difficult.

An issue that might provoke debate is the inconsistent and inaccurate methods used for biotype determination, including visual inspection and indirect measurements on dental casts or intraoral photographs. Usually, simple visual inspection is used in clinical practice and even in research to identify the gingival biotype. However, the accuracy of this method has never been documented. Egbhali et al^32^ reported that by using visual evaluation for gingival thickness, the biotype was accurately identified in about half of the cases, regardless of the clinician’s experience. As a result, other methods, such as direct measurements,33 gingival transparency,^34^ ultrasonic devices,^37^ and cone-beam computed tomography (CBCT)^35^ have been proposed.

Several studies have addressed the effects of different therapeutic methods on periodontal complications; gingival recession has been the main periodontal adverse outcome evaluated. Although this problem is not often attributable to the type of orthodontic appliance,^36,37^ there is debate in this regard. Some investigations have indicated that fixed appliances are associated with inflammation and even gingival recession,^38-41^ while some others have demonstrated no detrimental effects induced by the long-term presence of these appliances.^6,42^ However, it should be noted that these controversies might be due to the complex etiology of gingival recession, in which orthodontic appliances and fixed retainers are only two contributing factors.^43-47^ For example, thin soft tissues are more prone to the detrimental effects of environmental factors, such as plaque, calculus, and gingivitis;^13,17,48-50^ tooth position and alveolar bone anatomy might also play a role.^51^ In addition, there is insufficient evidence on some orthodontic parameters, such as force magnitude, location, and type of movement, which might result in dehiscence and gingival recession.^52^

Similar to biotype, different methods have been used to evaluate gingival recession. The measurement of the gingival recession on dental casts^29,53^ could be misleading due to factors, such as extrusion, crown fracture, attrition, or restoration.^54,55^ Crown length measurement for indirect estimation of gingival recession^8^ is also not reliable, as the tooth length might be affected by different factors, such as gingival hyperplasia.

Although some studies have demonstrated no significant association between malocclusion and biotype,^2,13,56^ some others have reported minimal gingival thickness in mandibular central and lateral incisors in class III patients.^5,30,57^ The periodontal tissue response has frequently been evaluated in class II patients, while it might be different in individuals with class III malocclusion. Six studies included in this review described the Angle classification for their participants, but only one^30^ clearly addressed the correlation between gingival biotype and the type of malocclusion.

One of the questions this review aimed to answer was the necessity of periodontal intervention for orthodontic patients with different gingival biotypes. As excessive labial inclination might lead to dehiscence and gingival recession on the labial surface,^47,59-61^ patients whose teeth are being moved labially (>95 degrees) should be informed about the risk of gingival recession.^17,58,62,63^ Therefore, bodily movement of the lower incisors should be preferred^2,64^ and exercised with caution in patients with thin gingival biotype and keratinized gingiva <2 mm.^11^ It has been indicated that as long as the orthodontic movements are confined to the alveolar process, the risk of periodontal lesions would be minimal.^50,65-69^

**Figure 2:**
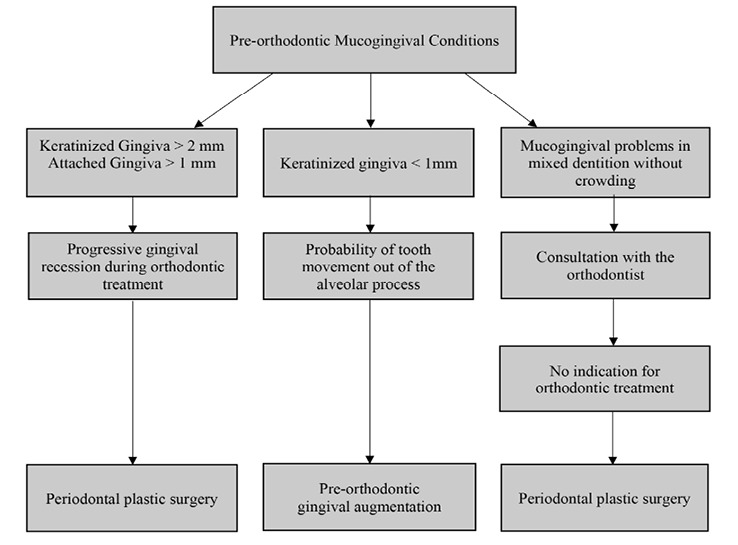
The management of pre-orthodontic treatment mucogingival problems.

**Figure 3:**
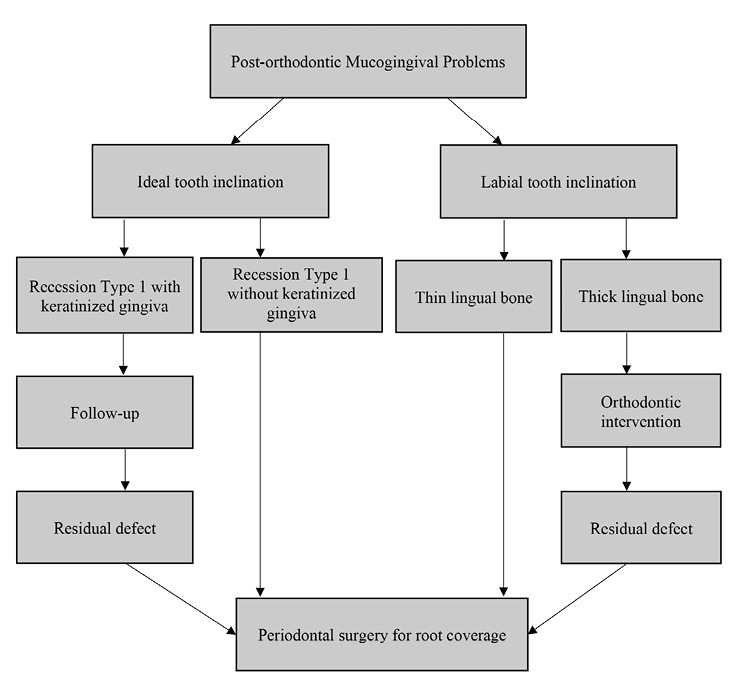
The management of post-orthodontic treatment mucogingival problems.

The width of the keratinized tissue plays a significant role in the decision-making process. It seems that periodontal intervention is rational in case of progressive recession or tooth movement out of the alveolar process (Figure 2). It should be noted that the recession (<2 mm) reported in some studies is usually not progressive and might be related to the heterogeneity of tissue quality.^55,70-72^ If gingival recession is observed after the orthodontic therapy, the treatment alternatives depend on its severity^73^ and the probability of elimination by orthodontic intervention (Figure 3). It should also be kept in mind that the clinical relevance of the induced recession is unclear, and most studies have only documented the pre- or post-orthodontic incidence of gingival recession.^7,36,60,74,75^

The strengths of this systematic review include the comprehensive search of the relevant literature, no restriction on the language or study design, and the quality assessment of the included studies.

## Conclusions

The existing evidence suggests that orthodontic treatment might result in small detrimental effects on the periodontium, especially in patients with thin gingival biotype. However, the available data do not make it possible to determine whether adverse periodontal changes are indicative of site-specific changes, host-specific factors, a direct consequence of the orthodontic forces, or study bias. It seems that due to the limited investigations and their inconsistent methodology, further well-designed prospective studies are necessary.

## Competing Interests

The authors declare no conflict(s) of interest related to the publication of this work.

## Authors’ Contributions

Design of the work: RA; acquisition of data: STD, MS, ADS, and MAS; analysis of data: RA, and AM; interpretation of data: RA, and MK; drafting the work: AM; revision: MK. All authors read and approved the final manuscript.
